# Good, Bad, and Ugly: Partner Support and Quality of Life Among Couples Facing Skin Cancer

**DOI:** 10.1093/geroni/igaa057.1390

**Published:** 2020-12-16

**Authors:** Laura Butner-Kozimor, Jyoti Savla

**Affiliations:** Virginia Tech, Blacksburg, Virginia, United States

## Abstract

When older adults in partnered relationships face a skin cancer diagnosis of one partner, couples may rely on one another for support. Previous studies have found that perceived support can influence one’s adjustment to the stressors associated with the skin cancer diagnosis, as well as influence the overall quality of life. Using dyadic data from 30 older couples (Mage = 70; SD = 7.25), this study examined positive and negative relationship-focused support strategies each partner provided and effects on the dyad’s quality of life. Dyadic path analyses simultaneously examined the impact of support received by one’s partner and its association with their own quality of life (actor effects) and their partner’s quality of life (partner effects). Positive support received by either partner, in the form of active engagement, was not associated with quality of life. In contrast, negative support in the form of protective buffering received from supporting partners was associated with poorer quality of life for themselves (β = -.37, p = .05) as well as for partners with skin cancer (β = -.43, p = .01). Similarly, overprotection, also a negative support strategy, by supporting partners was associated with poorer quality of life for partners with skin cancer (β = -.63, p < .001). Findings illustrate that not all types of support are beneficial for the overall couple relationship and couple outcomes. Implications for practice and interventions for older couples facing a cancer diagnosis will be discussed.

